# WE GOT THE MOVES LIKE JAGGER: REFLECTIONS ON AGING RESEARCH, EDUCATION, AND COMMUNITY

**DOI:** 10.1093/geroni/igae098.1246

**Published:** 2024-12-31

**Authors:** Joann Montepare

**Affiliations:** Lasell University, Newton, Massachusetts, United States

## Abstract

Over the last 50 years, tremendous advances have been made in our understanding of aging and its many components through lifespan developmental research. As well, educators in the field of aging have designed creative and impactful strategies for teaching about aging and dismantling ageist thinking. Together, scholars and educators have made age matter in meaningful ways in our contemporary world. This presentation uses examples from my professional experiences to reflect on several key aspects of aging research and education – and how community connections are necessary to sustain this work, with special attention to the need to advance age inclusivity in higher education.

